# Ipilmumab and cranial radiation in metastatic melanoma patients: a case series and review

**DOI:** 10.1186/s40425-015-0095-8

**Published:** 2015-12-15

**Authors:** Jonathan D. Schoenfeld, Anand Mahadevan, Scott R. Floyd, Michael A. Dyer, Paul J. Catalano, Brian M. Alexander, David F. McDermott, Irving D. Kaplan

**Affiliations:** Department of Radiation Oncology, Brigham and Women’s Hospital and Dana-Farber Cancer Institute, 450 Brookline Ave, DA L2-57, 02114 Boston, MA USA; Department of Radiation Oncology, Beth Israel Deaconess Hospital, Boston, MA USA; Department of Medical Oncology, Boston, MA USA; Harvard Radiation Oncology Program, Boston, MA USA; Department of Radiation Oncology, Duke Medical School, Durham, NC USA

**Keywords:** Melanoma, Ipilimumab, Immunotherapy, Radiation, Abscopal effect, Brain metastases, Stereotactic radiosurgery

## Abstract

**Background:**

Ipilimumab improves survival in metastatic melanoma patients. This population frequently develops brain metastases, which have been associated with poor survival and are often treated with radiation. Therefore, outcomes following ipilimumab and radiation are of interest, especially given case reports and animal studies suggest combined treatment may generate abscopal responses outside the radiation field.

**Findings:**

We reviewed sixteen consecutive melanoma patients who received 1 to 8 courses of radiation, with a sum total of 51, systematically evaluating abscopal responses by following the largest extra-cranial lesion. We also reviewed other series of patients treated with cranial radiation and ipilimumab.

Our patients received between 1 and 8 courses of cranial radiation. Four patients received radiation concurrently with ipilimumab. Median survival was 14 months, and 17 months in patients initially treated with SRS. Interestingly, after radiotherapy, there was a 2.8-fold increased likelihood that the rate of extra-cranial index lesion response improved that didn’t reach statistical significance (*p* = 0.07); this was more pronounced when ipilimumab was administered within three months of radiation (*p* < 0.01).

**Conclusion:**

Our experience and review of recently published series suggest ipilimumab and cranial radiation is well tolerated and can result in prolonged survival. Timing of ipilimumab administration in relation to radiation may impact outcomes. Additionally, our results demonstrate a trend for favorable systemic response following radiotherapy worthy of further evaluation in studies powered to detect potential synergies between radiation and immunotherapy.

## Findings

### Introduction

Approximately 30–50 % of metastatic melanoma patients develop brain metastases, which have historically been associated with limited survival of approximately four months [1]. Treatment typically includes whole-brain radiation (WBRT) or stereotactic radiosurgery (SRS) [2]. Ipilimumab has also demonstrated promise treating brain metastases in both a prospective trial [3] and a randomized trial including patients with intracranial disease [4].

Given the survival benefit associated with ipilimumab [4, 5] the combination of ipilimumab and cranial radiotherapy is of interest. Additionally, the effect of radiation and ipilimumab is intriguing given case reports and preclinical studies that suggest abscopal responses after radiation and immunotherapy [6, 7]. We sought to systematically explore this phenomenon by reviewing our experience and other recently published series describing patients treated with the combination of ipilimumab and cranial radiation.

## Materials and methods

### Patients and treatment

Sixteen consecutive patients with malignant melanoma were treated with ipilimumab and at least one instance of cranial radiation between 2008 and 2013 at our institution. This study was approved by our institutional review board as described in the Ackowledgments. No patient consent was required as this was a retrospective study.

Ipilimumab was generally given every three weeks for a total of four doses at a dose of either 3 mg/kg (*n* = 14) or 10 mg/kg (*n* = 2); patients achieving clinical benefit were offered maintenance therapy every twelve weeks. Cranial radiation was either WBRT or SRS. No patients received SRS as a planned boost. SRS was delivered using the Cyberknife (Accuray, Sunnvale, CA) system prescribed to the clinical tumor volume (CTV) which was equivalent to the planning tumor volume.

### Evaluation of response

A multimodality team including a medical, and radiation oncologist along with a neurosurgeon regularly followed all patients at least every 3 months until death. While receiving ipilimumab, evaluation of systemic response using mWHO criteria was performed with computed tomography or magnetic resonance imaging generally after four initial cycles and then at three-month intervals unless otherwise indicated. To evaluate abscopal effect, we extracted the longest diameter of the largest extra-cranial “index” lesion from the medical record, and changes between subsequent scans were calculated. A “delta-delta” was calculated as the difference in percent change of the index lesion on the two consecutive scans performed prior to radiation therapy as compared with the difference in the consecutive scans performed pre- and post-radiation as described previously [8].

### Statistical analysis

We calculated overall survival from the last day of the first cranial radiation course using the Kaplan-Meier method. Responses before and after radiotherapy were compared using McNemar’s and binomial tests for clustered data. Two-group exact tests for clustered data were used to explore the impact of treatment timing on response. All statistical tests were two-sided.

## Results

### Patient characteristics

Sixteen patients treated with radiation and ipilimumab received between one and eight courses of cranial radiation for a total of 51 courses (Table 1). Most patients (*n* = 10, 63 %) received four doses of ipilimumab; three patients received more than four doses as maintenance therapy, and three patients received less than 4 cycles because of disease progression. Median age at the time of first radiotherapy treatment was 57 (range 40 – 85 years). Of the 51 courses of brain-directed radiotherapy, 46 (90 %) were delivered by SRS. The remaining 5 radiation treatments were given as WBRT. WBRT was administered as the first radiation course in two patients who ultimately received subsequent SRS treatments, after previous SRS treatments in two patients, and in one patient who did not receive subsequent brain-directed radiotherapy. The median dose administered was 22 Gray (Gy, range 18 – 24 Gy) for all SRS treatments and 36 Gy (range 20–36 Gy) for all WBRT courses.

**Table 1:** Patient (*n* = 16) and radiation treatment (*n* = 51) characteristics

Patients	
Male sex	13 (81 %)
Age at time of initial brain radiation, median (range)	57 years (40 – 85 years)
Ipilimumab dosing	
10 mg/kg	2 (13 %)
3 mg/kg	14 (88 %)
Number of ipilimumab treatments, median (range)	4 (1 – 17)
Radiation Treatment	
Number of radiation courses per patient, median (range)	3 (1–8)
Number of lesions irradiated	
1	23 (45 %)
2-3	15 (29 %)
>3	13 (25 %)
Type of radiotherapy^a^	
Whole brain radiation	5 (10 %)
Stereotactic Radiosurgery	46 (90 %)
Radiation Dose (Gray), median (range)	
Whole brain radiation	36 Gy (20 – 36 Gy)
Stereotactic Radiosurgery^b^	22 Gy (18 – 24 Gy)
Location of index lesion	
Skin/subcutaneous tissue/lymph nodes	8 (26 %)
Lung	14 (45 %)
Other	9 (29 %)
None	3
Not imaged / Unknown	17

*Abbreviations*: *Gy* Gray

^a^Whole brain treatment given in 2 Gy fractions. Stereotactic radiosurgery treatments were administered in 1–5 fractions

^b^If multiple lesions were irradiated to different doses, the mean dose was used for tabulation

Seven patients received ipilimumab before brain-directed radiation, and 5 patients began ipilimumab after radiation. Four patients received radiation at some point while undergoing ipilimumab treatment and continued ipilimumab following radiation (concurrent treatment). Among these four patients who received concurrent treatment, in one patient, both treatments were given on the same day, and in two patients, concurrent treatment occurred multiple times. Overall, ipilimumab treatment was given within 3 months of radiation in 21 instances (41 %); in the remaining 59 %, ipilimumab was administered a median of 3.5 months prior to radiation.

### Outcomes

**Fig. 1:**
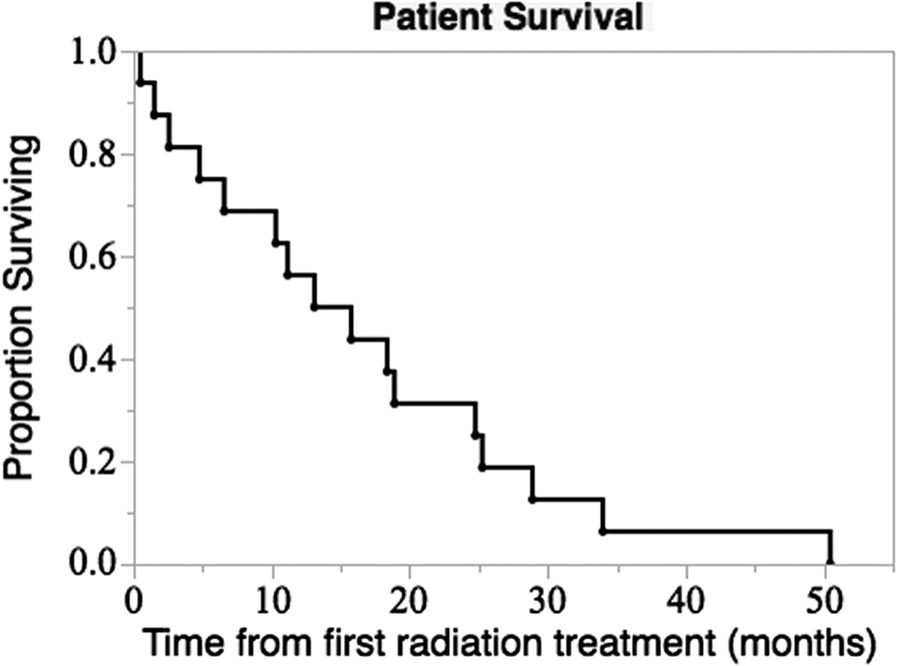
Patient Survival. Survival from first radiation treatment course (*n* = 16)

Median overall survival (OS) following first radiation treatment to the brain among all patients was 14.4 months, with a maximum OS of 50 months (Fig. 1). OS was 17 months in patients initially treated with SRS, and 19 months in patients that required multiple courses of cranial radiation. Patients who received SRS prior to initiating ipilimumab had superior OS as compared with patients who started ipilimumab prior to receiving SRS (median 26 months compared with 6 months, *p* < 0.001) despite similar age at diagnosis, number of lesions irradiated, number of cycles and dose of ipilimumab, and location of index lesion. Patients who received radiation concurrent with ipilimumab did not develop any significant immune related adverse events and had a median OS of 18 months.

Sequential imaging of systemic index lesions was performed before and after radiation in 31 instances. These lesions were most commonly located in the chest (45 %, Table 1). On the subsequent scan after radiation, in 11 of these 31 instances (35 %) the index lesion decreased in size as compared with the scan preceding radiation therapy. In comparison, in 25 of these 31 instances the index lesion size was also comparable on two scans prior to radiation treatment, and only 4 (17 %) demonstrated a favorable response (*p* = 0.20, McNemar’s test for clustered paired data). Among the 31 instances where imaging was available before and after radiation, there were 22 instances where two consecutive scans were also performed prior to the receipt of radiotherapy. In these cases, the response rate of index lesion response improved in 50 % of instances following radiation in comparison to worsening in 18 % (*p* = 0.07, binomial test for clustered data). In contrast, there was no significant association between time elapsed since first ipilimumab administration and favorable kinetics (*p* = 0.88).

**Fig. 2:**
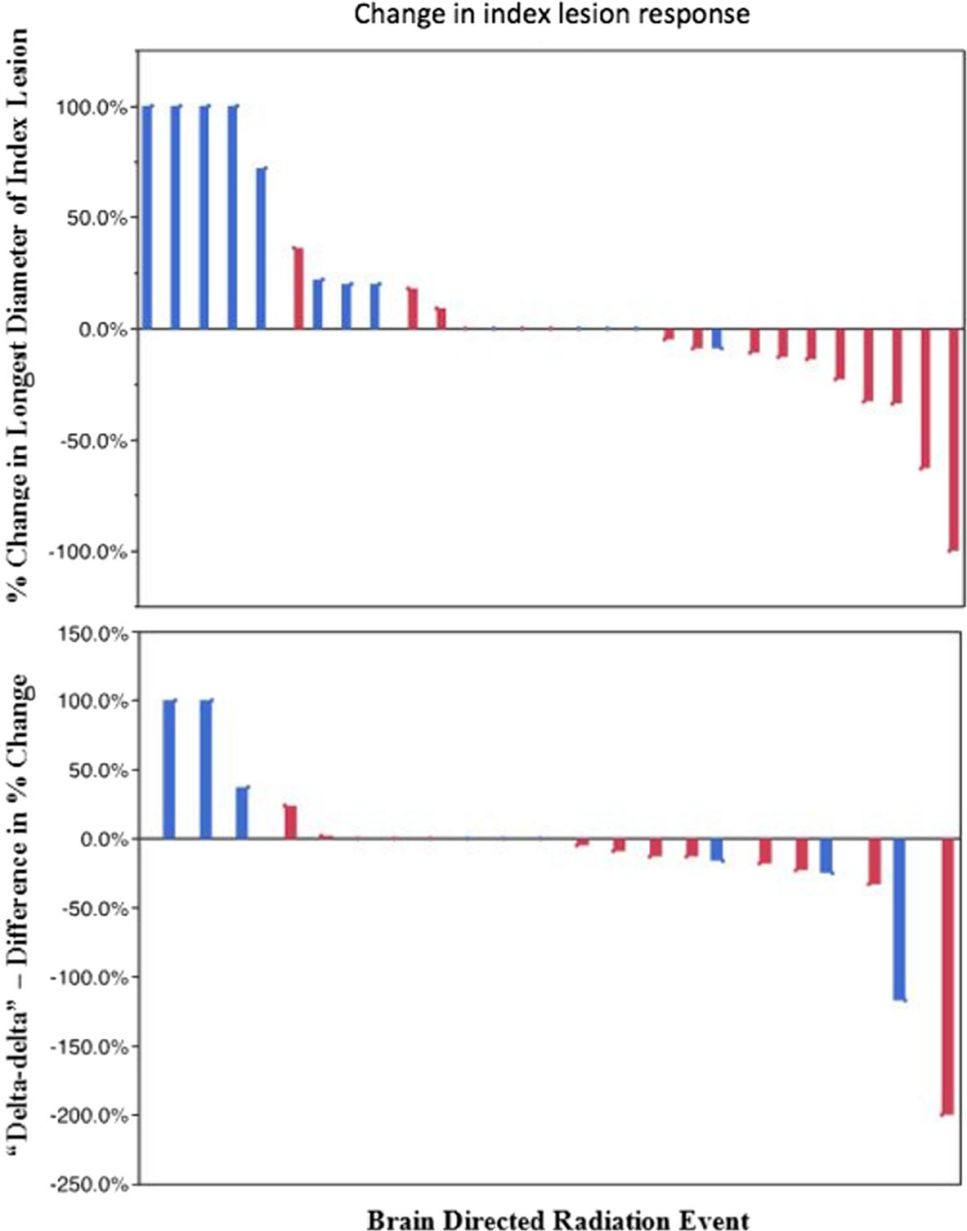
Change in index lesion response. Waterfall plots demonstrating index lesion responses for instances where sequential radiologic measurements were available. Both the percent change in the longest diameter of the index lesion after cranial irradiation (top, *n* = 29) and “delta-delta”, difference in percent change from the two imaging studies performed before cranial irradiation and before and after radiation (bottom, *n* = 22) are shown. Instances where ipilimumab was not given within three months of radiation are shown in blue, administration of both ipilimumab and radiation within a three-month span are shown in red

We examined the significance of the timing of ipilimumab in relation to radiation (Fig. 2). In the 31 instances where imaging was performed sequentially before and after radiation, index lesions were significantly more likely to decrease in size if ipilimumab was administered within the three months surrounding radiation treatment (63 % compared with 7 %, *p* = 0.003 by two-group exact test for clustered data).

## Discussion

We describe our experience treating melanoma patients with cranial radiation and ipilimumab. We report a median OS greater than 1 year in 16 patients that received a total of 51 radiotherapy courses including patients who received WBRT, multiple courses for recurrent disease, and patients who received radiotherapy concurrent with ipilimumab. These results compare favorably with historical series of melanoma patients with brain metastases treated with surgery and radiotherapy that describe median OS of less than 9 months, and less than 4 months in patients treated with radiotherapy alone [1]. Two-year OS in our series was 25 % and prolonged OS of over 50 months was observed. Similar favorable outcomes have also been seen in patients treated concurrently with ipilimumab and radiation of extra cranial lesions [9].

Review of the literature revealed 5 other recently published studies or abstracts that describe outcomes in 117 additional patients treated with the combination of cranial radiation and ipilimumab (Table 2). Ours is the only study to include patients initially treated with WBRT. None reported unacceptable rates immune related toxicity; however there are several reports of radionecrosis perhaps mediated in part by the immune system. Median OS in these studies ranged from approximately 5 months to almost 2.5 years, with favorable 1 and 2 year OS rates ranging from 40 − 67 %. The majority of these studies noted improved OS when SRS was administered prior to ipilimumab, as was observed in our study. In our study, baseline characteristics were similar between patients that received SRS prior or after ipilimumab; however, the limited numbers unfortunately precludes complex multivariate analysis. Although we were unable to adjust for unmeasured confounding variables, we currently find a strong association that would likely remain significant even if attenuated by other factors.

**Table 2:** Review of studies evaluating patients treated with cranial radiation and ipilimumab

	*N*	Median overall survival (mos)	Landmark survival	Steroid use	Timing issues	Radiation necrosis requiring surgery
Kiess et al. [10]	46	12.4	1 yr OS 40–65 % depending on timing of ipi	Routine use of ppx steroids	SRS before or during ipi associated with improved OS	5 patients
Knisely et al. [19]	27	21.3	2 yr OS 47 %	Not reported	SRS before ipi associated with trend towards improved survival	3 patients
Mathew et al. [11]	25	5^a^	6 mos OS 56 %	Routine use of ppx steroids	Not reported	0
Schoenfeld et al. (current study)	16	14.4	1 yr OS 54 %	No routine use of ppx steroids	SRS before ipi associated with better survival	0
Shoukat et al. [20]	11	28.3	1 yr OS 67 %	Not reported	Not reported	3 patients^b^
Tazi et al. [21]	10	29.3	2 yr OS 58 %	Not reported	All patients received SRS before or during ipi	Not reported

*Abbreviations*: *mos* months, *ppx* prophylactic, *OS* overall survival, *ipi* ipilimumab, *SRS* stereotactic radiosurgery

^a^Estimated from survival curve

^b^Use of surgery not specified

Interestingly, the two other studies that reported the shortest median OS reported routine use of prophylactic steroids at the time of SRS [10, 11]. Although data suggests that steroids administered for ipilimumab-induced immune-related adverse events do not adversely impact the efficacy of treatment, further evaluation of prophylactic steroids for asymptomatic patients may be warranted [12, 13].

Our analysis included evaluation of index systemic lesion response in relation to the timing of brain-directed radiotherapy and ipilimumab administration. Other retrospective studies have suggested radiation may be associated with a favorable systemic response in patients progressing on ipilimumab who receive radiation to a variety of sites [8, 14]. Similarly, we found that index lesions decreased in size after brain directed radiotherapy in 63 % patients who received both radiotherapy and ipilimumab within a three-month span. The limited number of patients analyzed constrained our power to detect statistically significant differences; however index lesions were approximately twice as likely to decrease in size on imaging following radiation, with the additive benefit of improved kinetics of response. Although delayed index lesion response to ipilimumab or favorable response to ipilimumab as compared to other systemic agents may partially explain these results, ipilimumab had been administered for more than three months prior to radiotherapy in 27 (53 %) of instances and there was no significant association between duration of ipilimumab and favorable kinetics.

Radiation has potential immunogenic properties [15, 16] and the combination of ipilimumab and local radiotherapy increased systemic anti-tumor responses and improved OS in a mouse model of breast carcinoma [17]. Case reports and small studies have suggested similar radiation-stimulated immune phenomenon in humans, with the combination of radiation and ipilimumab resulting in increased antigen targeting, decreased numbers of inhibitory myeloid-derived suppressor cells, and increased levels of anti-tumor antibodies and circulating activated T-cells [6, 7, 18]. Our results add to this growing body of evidence that suggest that future studies should continue to examine the potential synergies between radiotherapy and immunotherapy.

## Abbreviations

WBRT: Whole brain radiation therapy
SRS: Stereotactic radiosurgery
Ipi: Ipilimumab
OS: Overall survival
